# Neural Decoding of Visual Information Across Different Neural Recording Modalities and Approaches

**DOI:** 10.1007/s11633-022-1335-2

**Published:** 2022-07-14

**Authors:** Yi-Jun Zhang, Zhao-Fei Yu, Jian. K. Liu, Tie-Jun Huang

**Affiliations:** 1 Department of Computer Science and Engineering, Shanghai Jiao Tong University, Shanghai, 200240 China; 2 School of Computer Science, Peking University, Beijing, 100190 China; 3 Institute for Artificial Intelligence, Peking University, Beijing, 100190 China; 4 School of Computing, University of Leeds, Leeds, LS2 9JT UK; 5 Beijing Academy of Artificial Intelligence, Beijing, 100190 China

**Keywords:** Neural decoding, machine learning, deep learning, visual decoding, brain-inspired vision

## Abstract

Vision plays a peculiar role in intelligence. Visual information, forming a large part of the sensory information, is fed into the human brain to formulate various types of cognition and behaviours that make humans become intelligent agents. Recent advances have led to the development of brain-inspired algorithms and models for machine vision. One of the key components of these methods is the utilization of the computational principles underlying biological neurons. Additionally, advanced experimental neuroscience techniques have generated different types of neural signals that carry essential visual information. Thus, there is a high demand for mapping out functional models for reading out visual information from neural signals. Here, we briefly review recent progress on this issue with a focus on how machine learning techniques can help in the development of models for contending various types of neural signals, from fine-scale neural spikes and single-cell calcium imaging to coarse-scale electroencephalography (EEG) and functional magnetic resonance imaging recordings of brain signals.

## 1 Introduction

Every day, various types of sensory information from the external environment are transferred to the brain through different modalities and then processed to generate a series of coping behaviours. Among these perceptual modalities, vision is arguably the dominant contributor to the interactions between the external environment and the brain. Approximately 70 percent of human perception information is derived from vision[1], far more than the auditory system, tactile system, and other sensory systems combined. The visual system is the part of the central nervous system that is required for visual perception, processing, and interpreting visual information to build a representation of the visual environment. It consists of the eye, retina, fibers that conduct visual information to the thalamus, the superior colliculus, and parts of the cerebral cortex. Today, researchers can collect neural signals using different recording modalities, e.g., spikes, electroencephalography (EEG), and functional magnetic resonance imaging (fMRI), from brain activity in different parts of the visual system, such as the retina, lateral geniculate nucleus (LGN), and primary visual cortex (V1) cortex, etc. Depending on the corresponding collecting devices, different recording modalities differ in their invasiveness, scale, and precision.

Neural coding is an important topic for understanding how the brain processes stimuli from the environment[2, 3]. The aim of neural decoding is to read out information embedded in various types of neural signals[4]. As for vision, understanding how neurons perceive and respond to rich natural visual information is a major topic of neural encoding[5, 6], whereas, the goal of neural decoding of visual information is to restore the original stimulus from neural responses as much as possible[7], as shown in Fig. 1. It is also critical to the development of artificial vision used by brain-computer interfaces and virtual reality devices.

Much effort has been made to study the various mechanisms underlying neural decoding in the visual pathway in recent decades[8–12]. These mechanisms can be roughly divided into three categories depending on the decoding type: 1) Visual stimulus classification, in which a specific

Review
Special Issue on Brain-inspired Machine Learning
Manuscript received February 7, 2022; accepted April 27, 2022; published online July 14, 2022
Recommended by Associate Editor Chun-Hua Shen
Colored figures are available in the online version at https://link.springer.com/journal/11633
© The Author(s) 2022

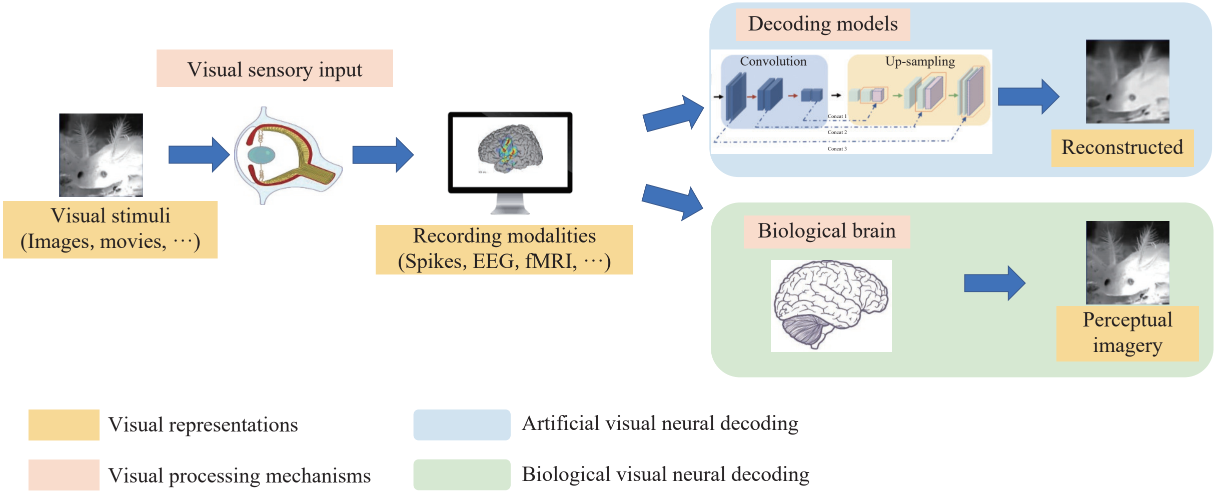

Fig. 1 Illustration of two types of visual neural decoding stimulus is classified into the best-matched image set; 2) Visual stimulus identification, in which the stimulus is identified with a specific visual object; 3) Visual stimuli reconstruction, in which the corresponding visual stimulus is reconstructed in accordance with the resulting neural responses. Most decoding approaches have depended on linear methods due to their interpretability and computational efficiency[9, 13, 14]. Although linear decoding methods are capable of decoding spatially uniform white noise stimuli and the coarse structure of natural scene stimuli from neural responses[13, 14], the recovery of the fine visual details of naturalistic images is difficult for these types of methods. The most recent decoders utilized nonlinear methods for the fine decoding of complex visual stimuli. For instance, optimal Bayesian decoding was leveraged for white noise stimuli, but achieved limited generalizability to a large neural population[15]. For natural scene image structures, key prior information was used to perform computationally expensive approximations to Bayesian inference[16, 17]. Some researchers have combined linear and nonlinear approaches to generate coarse reconstructions of natural stimuli through calcium imaging data[10, 18–20]. Additionally, many researchers have begun to successfully use deep learning techniques for visual neural decoding, leading to the great achievement in artificial vision[11, 21–24].

Visual neural decoding is a significant issue that can help advance engineering applications such as brain–machine interfaces and a more holistic understanding of the brain in neuroscience. Considering the rapid developments of related techniques in visual neural decoding, there is a strong demand for a comprehensive and up-to-date review in this field. In this review, we sorted out the research evolution in visual neural decoding. Various neural recording modalities are introduced in this review, especially for the emerging calcium imaging data. We summarized the advantages and disadvantages of different neural decoding methods. In addition, open resources, including public neural data and software toolkits, are also provided for the convenience of neural decoding research. Finally, we conclude with our perspective on the open challenges and future directions for the outlook in this study. We aim to provide a review of neural decoding in visual systems that could serve as an inspiration to both neuroscience and multidisciplinary researchers looking to understand the state-of-the-art and current problems in neural decoding, especially regarding the development of artificial intelligence and brain-like vision systems.

## 2 Task evolution in visual neural decoding

Visual neural decoding has been a core topic in computational neuroscience in recent decades. To date, the history of visual neural decoding has involved several stages from the perspective of decoding tasks. Advancements in recording devices of different modalities and increasing efforts in decoding research have allowed more challenging tasks that have been accomplished with researchers′ endeavors. Visual neural decoding methods can be coarsely divided into three categories: image classification, image identification, and image reconstruction. In image classification, the stimulus that evoked the neural response recording is classified into a specific stimulus set comprised of similar stimuli. For example, Kamitani and Tong[25] successfully predicted which of the eight stimulus orientations the subject was seeing from individual fMRI signals recording trials. Yargholi and Hossein-Zadeh[26] used an augmented naïve Bayesian classifier to decode the fMRI signals generated in response to handwritten digits into correct classes. In image identification, one stimulus is identified as the specific image (usually the most similar image) according to the corresponding response; this category is seen as more challenging than classification. Kay et al.[27] proposed receptive-field models for different voxels that allowed the identification of the specific image the subject was seeing from a large set of completely novel natural images. Horikawa and Kamitanin[28] identified the object seen in the most similar category according to a feature vector predicted by measured fMRI activity. Image reconstruction typically involves methods that perform pixel-level reconstructions of stimuli from the recorded responses, which is evidently more difficult than the above two decoding tasks and also the recent focus of neuroscience researchers. The deep learning neural network (DNN) for visual decoding is the most frequently proposed approach to solve the reconstruction problem in recent years[12].

Image classification and identification have been forms of decoding tasks in the early stages of development in the field of neural decoding. In recent years, researchers in the field of neural decoding have mainly regarded reconstruction as the task goal of neural decoding (see Section 4). In order to reflect the latest developments in the field of neural decoding, in this review, we mainly focus on the reconstruction task in neural decoding. Except for special emphasis, the literature and methods mentioned in this paper are generally oriented toward reconstruction tasks of neural decoding. Those emerging decoding tasks beyond reconstruction will be discussed in future directions in Section 6.

## 3 Neural recording modalities

As the input to various types of neural decoders, it is important to understand the characteristics of the signals obtained with neural recording modalities, as shown in Fig 2. The differences in signal types, data structures, and spatial and temporal resolutions, which are summarized in Fig. 3, have a great influence on the design of visual neural decoders.

### 3.1 Spikes

Action potentials are fast electrical changes that are triggered when the membrane potential of individual neurons depolarizes their thresholds that can be measured as consistent waveforms (called "spikes")[29]. Spike events are usually detectable with microelectrodes or microelectrode arrays, with a high temporal resolution decoding on a millisecond timescale. Spikes are the most common invasive recording methods, in which electrodes are inserted into or adjacent to neurons to record voltage events[30−32]. Because a large number of electrodes need to be implanted in parallel into a single probe in order to record a large number of neurons simultaneously, the neural information collected from subjects depends on the sophistication of the underlying electrophysiology technology[33, 34].

In certain neural decoding systems based on spike signals, spike events are usually converted to firing rates (in a fixed time bin), as shown in Fig. 3, which are then fed into neural decoders[23]. The temporal coding of spike events is rarely seen in neural decoding models based on spike signals. Some dimensionality reduction methods are used prior to decoding when a large number of neurons are recorded prior to decoding[35−37]. Such dimensionality reduction methods range from the classical machine learning methods, e.g., principal component analysis, to deep learning algorithms.

Recent advances in neural decoding with spike signals collected from visual systems have benefitted from relevant recording technology, such as Neuropixels probes and the increased availability of public datasets[1, 38−41]. Zhang et al.[23] used spike signals collected from salamander retinas to recover the dynamic movie stimuli. Iqbal et al.[42] used a deep neural network to decode natural stimuli from spike responses collected in the mouse cortex. Leveraging the development of large-scale multielectrode recording systems, Kim et al.[43] applied the spike signals of 2 000 retinal ganglion cells to develop a multistage decoding approach that exhibited improved accuracy over linear methods. Xu et al.[44] proposed a deep spike pattern decoder, which was not only capable of perceiving inputs from noisy environments, but also had a good reconstruction ability of generalizability to images, fMRI brain activities, and sound signals.

Spike signals are still the most widely used neural

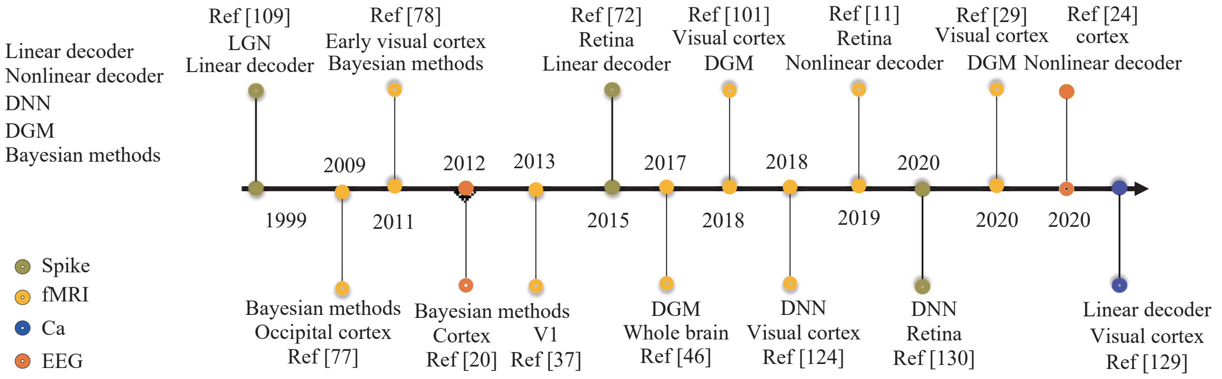

Fig. 2 Key methods in the history of visual neural decoding. On the left, different colored circles represent different neural recording modalities. The decoding methods are indicated with different colored text.

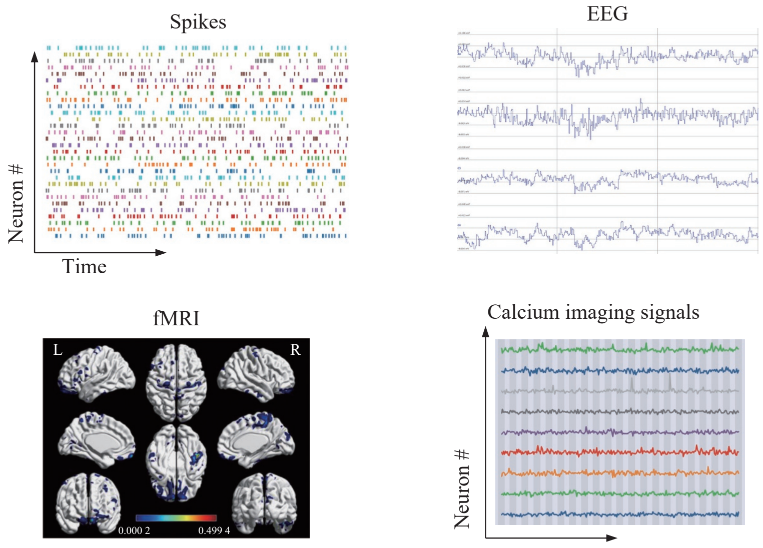

| | Advantages | Shortcomings | Spatial/ Temporal resolution |
|---|---|---|---|
| Spikes[61] | Single-cell resolution; High temporal resolution | Highly invasive | 1–10 μm 0.5–4 ms |
| EEG[57,70] | Non-invasive | Hard to localize signal source | 5–10 mm 10 ms–10 s |
| fMRI[59] | Non-invasive; Global brain recording | Low temporal resolution; Indirect recording of brain activity | 1–10 mm 1–20 s |
| Calcium signal[18] | Single-cell resolution; Less invasive than spike | Invasive Indicator-sensitive | 100 μm 30 ms |

Fig. 3 Multiple recording neural modalities data in visual neural decoding, and consequently, studies on visual neural decoding based on spike signals are predominant in this field. Signals from other recording modalities can be transferred to spike signals first and then decoded with appropriate neural decoders designed for spikes. For example, in some visual decoding studies, calcium imaging traces are converted to spike events with transcoding algorithms, which are then decoded to extract stimulus information (see Section 3.4).

## 3.2 EEG

EEG is a low-cost, non-invasive neuroimaging technique that provides high temporal resolution recordings of brain activity, and consequently, has been widely applied in various fields, e.g., brain-computer interface systems and brain activity monitoring. However, EEG has limitations resulting from the recording technique. As illustrated in Fig. 3, the complex, high-dimensional EEG signals usually have a low signal-to-noise ratio and nonlinearity and nonstationary properties, resulting in signals that require artificial removal[45−48].

Due to the complexity and information redundancy of EEG, one critical component of EEG neural decoding is the extraction of features from the original EEG signals. Researchers have proposed a series of feature extraction methods that operate from different aspects of the signal. For example, methods focusing on the time-domain aspects of the signal include independent component analysis and autoregressive models; those investigating frequency-domain information from EEG signals include fast Fourier transform and Welch's method; and wavelet transform and short-time Fourier transform can be used to conduct time-frequency domain analyses. Recent studies have analyzed the advantages and shortcomings of each feature extraction method, noting that the appropriate feature extraction method must be chosen according to the specific type of task[49−53].

One general decoding goal of EEG acquisition is classification. The EEG classification process pipeline involves data preprocessing and splitting the dataset into a training set for training the classifiers and a test set for predicting the new data classes. In addition to convention algorithms, e.g., support vector machine and linear discriminant analysis, various machine learning and deep learning algorithms have been used for EEG classification tasks, e.g., motor imagery processing, motion recognition and attention disorder classification[54−56]. Beyond these fields, various computational neural decoding models have been proposed[57−60]. Garofalo et al.[61] found that the event-related potential signals of prediction errors are modulated by the likelihood of occurrence based on the predicted response-outcome model.

In addition to the classification decoding tasks, EEGs have been proposed for use with inverted encoding models which attempt to reconstruct the contents of memory or attentional focus of EEG brain activity[62–66].

### 3.3 fMRI

fMRI is the most frequently used non-invasive recording modality, in human decoding experiments. As shown in the fMRI recording of Fig. 3, blood oxygenation is measured by fMRI and utilized as a proxy of neural activity. Moreover, fMRI allows the simultaneous recording of whole-brain blood oxygenation-level dependent activity, providing the largest scale coverage among all recording approaches as well as insight into brain function study. fMRI represents the recorded signals in different "voxels" (locations) of the brain during the experiment[67, 68].

Univariate methods were typically used for fMRI brain activity pattern analysis in the early stage, employing a general linear model that was used to estimate each voxel in the brain separately in most univariate methods[69]. However, Univariate methods depend on a uniform relation between neural activity and the investigated function, in both individual voxels and across participants, which makes it difficult to detect spatial patterns[70]. To overcome this limitation, multivariate pattern analysis was proposed, capable of detecting the activation distribution of the brain and accurately decoding the cognitive state of the patient[71]. Thus, multivariate methods are widely used to train classifiers on unsmoothed, voxelwise, patterns of brain activity in different conditions.

In addition, some researchers used their own handcrafted methods to process fMRI data in visual decoding. For example, Zhang et al.[23] unravelled the voxel data as a one-dimensional vector to input them into a deep neural network decoder. Du et al.[72] assumed that the correlations among fMRI voxels could naturally reflect the characteristics of the corresponding visual stimuli and used a full-covariance matrix to capture these correlations in their decoding experiments.

### 3.4 Calcium imaging signal

Calcium imaging is another invasive technique for recording the activity of activated neurons[73]. In calcium imaging data, neuronal activity is measured by the fluorescence intensity of calcium indicators, which is captured by an electron microscope; these calcium indicators are usually chosen according to the type of neuron. As the traces are shown in Fig. 3 calcium signals part, the raw outputs of two-photon electron microscopes in the calcium imaging experiment are videos, which record the locations and fluorescence changes of activated neurons during the measuring progress[74]. Usually, traces of fluorescence changes in calcium indicators are extracted from the recording video for further analysis[75].

Visual decoding from calcium imaging data is a relatively recent and understudied field in visual information processing. Nevertheless, several methods have been proposed by calcium imaging researchers. Previous work has focused on classification algorithms. For example, Grewe et al.[78] attempted to use four machine learning structures to classify the V1 responses of natural stimuli according to the calcium traces acquired during the selected frames. A series of studies converted calcium imaging traces to spike events[75–81], and their findings have been leveraged to conduct several neural decoding studies based on calcium imaging data[19, 20]. For example, Garasto et al.[19] conducted a pixel-by-pixel reconstruction of a complex natural stimulus from the spike count estimated from the calcium imaging response of the mouse primary visual cosrtex (V1). In some recent work, the standard ratio of fluorescence change (usually depicted as $\mathrm{d}F/F_0$ form) of each of the regions of interest (ROIs) during a blank screen prior ($F_0$) to stimulus presentation ($F$) was calculated as the neuron's calcium imaging response. Tang et al.[82] evaluated how well the sparse calcium imaging response allowed a decoder to discriminate 2 250 natural stimuli. Yoshida and Ohki[10] believed that natural images could be reliably reconstructed by sparse calcium imaging population responses in the mouse visual cortex.

## 4 Decoding approaches

As illustrated in Fig. 1, the visual neural decoding problem can be formulated as the identification of stimulus $x=(x_1,\cdots,x_N)^{\mathrm{T}}$ that produces the observed brain activity $y=(y_1,\cdots,y_M)^{\mathrm{T}}$ best. The stimulus in visual neural decoding tasks is usually presented as an image, one movie frame, etc. Neural decoding specifically refers to predicting $x$ from $y$, in which the representation of the external environment in neural recordings is prospectively revealed.

### 4.1 Linear decoding methods

In recent decades, various methods have been developed. Fig. 2 illustrates a timeline of some key studies in the history of visual neural decoding. Traditionally, a neural decoder can be optimized with linear and nonlinear statistical methods[8–11]. In linear decoding methods, the relationship between stimulus set $X$ and neural response set $Y$ can be formulated as follows:

$$X=YW. \tag{1}$$

The optimal weight matrix for the linear decoding model $W$ is calculated using least squares regression:

$$W_{op} = \left(Y^{\mathrm{T}}Y\right)^{-1} Y^{\mathrm{T}}X. \tag{2}$$

And the weights $W_{op}$ are then used to reconstruct the stimulus in a held-out set of brain activity datasets.

For instance, Schoenmakers et al.[83] reconstructed BRAINS characters from measurements of human brain activity using the linear reconstruction approach. Linear decoding methods are derived from the reverse of early-stage linear encoding models in neuroscience. Due to the limited representation power of these structures, the decoding tasks were usually simple and the performance in natural image reconstructions was not satisfactory. To overcome this limitation, Brackbill et al.[14] combined nonlinear and linear reconstructions to develop a cascade decoding model for retinal ganglion cell (RGC) natural stimulus-response signals.

Most early approaches to visual neural decoding have depended on linear methods due to their interpretability and computational efficiency. Nevertheless, the limited representation power of linear methods makes it difficult to reconstruct the fine visual details of natural images.

## 4.2 Bayesian-based decoding methods

More recent visual decoding studies have incorporated nonlinear methods for better reconstruction performance with complex natural stimuli, as illustrated in Fig. 2. Among these studies, a series of Bayesian methods have been proposed to explore the correlations between neural recording signals and visual stimuli.

In a typical Bayesian-based method, the visual neural decoding problem can be formulated as the following maximum a posteriori probability (MAP) estimation problem:

$$\hat{\boldsymbol{x}} = \arg\max_{\boldsymbol{x}} p(\boldsymbol{x} \mid \boldsymbol{y}). \tag{3}$$

Here, $\hat{\boldsymbol{x}}$ is the best matching stimulus among all $\boldsymbol{x}$. $\boldsymbol{y}$ represents the recorded brain activity signals. Specifically, the stimuli $\boldsymbol{x}$ can be then presented as images or movie frames, for example. The interpretation of modality $\boldsymbol{y}$ depends on the specific neural recording technique used in the physiological experiment. Typically, for the sake of simplification, both the stimulus and the response are assumed to be zero mean, standard Gaussian distributions. In practice, let the acquired dataset $\mathcal{D} = \left\{\left(\boldsymbol{x}^{(t)}, \boldsymbol{y}^{(t)}\right)\right\}_{t=1}^{T}$ consists of $T$ stimulus-response pairs. Then, by plugging in the predictive density, the MAP solution can be formulated as

$$p(\boldsymbol{x} \mid \boldsymbol{y}, \mathcal{D}) = \int_{\boldsymbol{\theta}} p(\boldsymbol{x} \mid \boldsymbol{y}, \boldsymbol{\theta}) p(\boldsymbol{\theta} \mid \mathcal{D}) \tag{4}$$

and further simplified to $p(\boldsymbol{x} \mid \boldsymbol{y}, \mathcal{D}) = p(\boldsymbol{x} \mid \boldsymbol{y}, \hat{\boldsymbol{\theta}})$, where $\hat{\boldsymbol{\theta}} = \arg\max_{\boldsymbol{\theta}} p(\boldsymbol{\theta} \mid \mathcal{D})$. Notably, in practice, many unique stimuli in $\mathcal{D}$ are used to sample the stimulus space accurately, and the average value of responses to the same stimulus is used as one of the $\boldsymbol{y}$ values to weaken the influence of noise.

Naselaris et al.[16] presented a Bayesian framework for accurate reconstructions of the spatial structure of natural images. However, in their framework, a reconstruction was defined as the image with the highest posterior probability that was thought to evoke the corresponding brain activities. Nishimoto et al.[17] constructed a Bayesian decoder with a sampled natural movie prior to reconstructing a movie stimulus from fMRI signals. In their framework, a motion-energy encoding model was presented to match the slow fMRI responses to dynamic stimuli.

Gallant first used a Bayesian reconstruction model to decode fMRI data from early visual areas, and the resulting reconstruction is a predefined natural image, selected according to its posterior probability[16, 17, 84]. Fujiwara et al.[84] proposed a Bayesian canonical correlation analysis in which image bases were automatically learned, and an invertible mapping was obtained between brain activity and image bases. Recently, Du et al.[72] leveraged the latent variables inferred by Bayesian reconstruction to capture the correlations among the voxel activities of fMRI signals; this resulted in an increased reconstruction accuracy.

As mentioned above, Bayesian decoding models usually outperformed simple linear decoding models in visual neural decoding tasks. However, there are also some constraints for Bayesian decoding methods. The Bayesian decoding methods usually have to resort to the specific prior information encoded by a specifically designed model. The determination of parameters in the overall decoding process needs to be elaborated. Furthermore, the mapping between the visual stimuli and the corresponding brain activity determined by Bayesian methods does not typically describe the relationship between these two cross-modal data. Consequently, fine natural image details are difficult to be reconstructed with this type of method.

## 4.3 Deep neural network methods

As illustrated in Fig. 2, deep learning techniques, especially deep neural networks, have been implemented in the neural decoding field in recent years. Some studies have revealed that the essential DNN mechanism corresponds to the human visual cortex[28, 85–87]. Some designs of essential blocks in deep learning have been inspired by developments in neuroscience. For example, the concept of simple and complex cells in the V1 primate cortex has inspired computation model development from a micro perspective[88, 89], e.g., the popular convolutional neural network (CNN) submodule in deep learning. DNN models usually comprise simple modules, e.g., simple artificial neurons running matrix multiplication and nonlinearity computations, and complex layered modules, e.g., convolutional neural network CNNs, recurrent neural networks (RNNs) [90–95].

CNN is the most popular module in various deep learning neural network structures. The core idea of the CNN was derived from the visual cortex of cats by Hubel and Wiesel[96]. Specifically, CNN uses convolutional layers with convolutional kernels (analogous to receptive fields in neuroscience) of different sizes to extract visual features from the input image layer by layer[91, 92, 95, 97]. Simplistically, the 2-dimensional image convolution operation can be written as follows:

$$\text{filter } 2d = \sum_{i=0}^{k}\sum_{j=0}^{k} g(i,j) \otimes X(k-i, k-j) \tag{5}$$

where $X$ is the input image matrix to be convolved with the kernel $h$. The indices $i$ and $j$ depend on the image size, and $k$ depends on the kernel size. Usually, 3-dimensional convolution is conducted in a CNN, with an additional dimension called channel, i.e., 2D convolutional operations with different kernels. Other layers also contribute to the benefits of CNNs in the computer vision field, such as pooling layers that reduce the size of features fed to the next layer for computational efficiency, dropout layers that ameliorate overfitting by randomly closing neurons during training, and softmax layers that help score the classification problem. Recent advances in CNN algorithms have led to outstanding performance in various tasks, such as image processing and natural language processing[98–101]. Even so, the performance of the CNN is highly dependent on hyperparameter tuning, such as the number of layers and the kernel sizes in the convolutional layers.

The RNN is a time series-based deep learning algorithm that utilizes sequential data with temporal information from network inputs, in contrast to traditional deep neural networks, which regard the input and output as independent[98]. The output of the RNN depends on the prior outputs and the current inputs. Generally, the general formulation of RNN can be formulated as follows:

$$\begin{aligned} \text{net}_t &= Ux_t + Ws_{t-1} \\ s_{t-1} &= f(\text{net}_{t-1}) \end{aligned} \tag{6}$$

where $x_t$ is the input at time $t$ and $s_{t-1}$ is the output of the RNN at time $t-1$, $U$ and $W$ are their corresponding weights. $f(*)$ is a nonlinear operation. Many variants of the RNN have been proposed for specific tasks, e.g., classic long short-term memory (LSTM)[93], gated recurrent units[102], and multiplicative LSTM[103]. RNN architectures have been applied in many fields due to their effective time-series data analysis characteristics, such as speech recognition[104], natural language processing[105], and signal identification[106]. Given the essential temporal characteristics of neural recording data, there are exciting prospects for applying RNNs in visual neural coding. Even for the retina, it was shown that the role of recurrence is important for encoding of dynamics videos[107].

Generative adversarial networks (GANs) are another popular approach used for visual neural decoding. Goodfellow et al.[92] first proposed the core mechanism of the GAN from game theory, i.e., the generator network attempts to generate data that can fool the discriminator network, while the discriminator network attempts to distinguish the generated (forged) data from real data[108], which can be briefly formulated as follows:

$$\begin{aligned} \min\max V(D,G) = &E_{x\sim p_{\text{data}}(x)}[\log D(x)]+ \\ &E_{z\sim p_z(z)}[\log(1-D(G(z)))] \end{aligned} \tag{7}$$

where $D$ and $G$ are the discriminator and generator, respectively, $\sim p_{\text{data}}$ is the distribution of the real data, $p_z$ is the distribution of noise, and $E$ is the expectation. Here, the generative model captures the distribution of the real data and is trained to maximize the probability that the discriminator will make a mistake. The generator is trained while the discriminator is idle to obtain its predictions when attempting to fool the discriminator. Such steps are repeated as both the discriminator and generator improve in their respective tasks. Various types of GANs have been proposed: Conditional GAN (CGAN)[108], deep convolutional GAN (DCGAN)[109], Laplacian pyramid GAN (LAPGAN)[110], and superresolution GAN (SRGAN)[111].

Transfer learning is an emerging technique in machine learning[112, 113] in which a machine exploits the knowledge gained from a previous task to improve the generalizability of another[114, 115]; usually, the scale of data in the original training task is larger than that in the new problem. In this case, the advantages of transfer learning are obvious, i.e., it reduces training time, results in better neural network performance (in most cases), and does not need much data. The neural coding issue is quite appropriate for transfer-learning applications, given the scarcity of neural recording data. Indeed, a recent study shows that a pre-trained DNN decoder can be used for real-time video decoding via spikes[116]. The use of transfer learning techniques in visual neural decoding is discussed in Section 6.

Although conventional visual neural decoding methods, involving the application of linear decoding and simple nonlinear decoding approaches, have made achievements in the visual neural decoding of white noise and simple artificial pattern stimuli, these methods have encountered a bottleneck in regard to natural scene stimulus decoding. Meanwhile, the great representation power of deep learning techniques has attracted more and more researchers to apply them in various research fields, including neuroscience.

In the last several years, DNN-based neural decoding methods have been proposed to address this task. For example, Parthasarathy et al.[22] first linearly reconstructed an image from simulated RGC spike signals and then enhanced it through a deep neural network. The reconstruction performance with their decoding framework outperformed that of linear decoding methods. To overcome the limitations of Parthasarathy′s methods, Zhang et al.[23] proposed an end-to-end decoding framework and reconstructed dynamic movie stimuli from salamander RGC signals. Shen et al.[117] designed an end-to-end direct reconstruction model in which fMRI brain signals are decoded to natural stimuli. Furthermore, Shen et al.[118] explored the generalizability of their decoding model from natural stimuli to artificial images.

Apart from the supervised learning DNN methods (in which the ground truths for the reconstruction targets are provided) above, as illustrated in Fig. 2, recent studies have also focused on the application of deep generative techniques in visual neural decoding, for example, the variational autoencoder (VAE). Advancing beyond pairwise matching (stimulus-response) for a given real physiological dataset, the VAE uses an encoder to describe the probability distribution of the latent state space. New stimuli that do not exist in real physiological datasets can be created by sampling from the latent state space, resembling the regular patterns of real stimuli. Han et al.[119] reconstructed the video inputs of the fMRI activity by converting the latent variables to the video frames through the VAE′s decoder. Du et al. proposed a neural decoding model based on the VAE to learn the disentangled image representations. Furthermore, Du et al.[72] first introduced the Bayesian deep learning to a neural decoding study and combined it with a multiview deep generative model called DGMM.

Due to the great success of GANs in synthesizing high-fidelity images, some researchers have leveraged GAN-based approaches to generate visual stimuli. St-Yves and Naselaris[120] trained their CGANs with reconstructed images conditioned on given fMRI signals. Gerven et al.[121] trained a DCGAN separately on image datasets to learn the latent state space and then used it to generate handwritten characters and natural grayscale images from fMRI signals. Some GAN-based methods have been proposed for human face image decoding. For example, Gerven et al.[122] first inverted the linear transformation from latent features to neural responses with MAP estimation. Then, adversarial training was used to perform a nonlinear transformation from perceived stimuli to latent features. VanRullen and Reddy[123] trained a VAE using a GAN over a large celebrity face dataset and obtained the corresponding latent space. When linear-transformed fMRI signals are input to the VAE, both robust pairwise decoding and accurate gender classification can be achieved. Compared with linear reconstruction methods, nonlinear methods, especially DNN-based methods, can greatly improve the accuracy of natural image reconstruction, especially for visual details. Although the performances of current DNN-based decoding methods depend greatly on the scale of neural data, deep learning techniques are still one of the most promising methods for the development of visual neural decoding.

## 5 Open resources

The open-source nature of datasets with high-quality neuronal physiological responses is essential for neuroscience research. These open-source data objectively connect different research works through sharing data and play an important role in forming benchmarks in the field of neural decoding. Here, we summarize three large-scale open neural databases widely used worldwide, as well as some neural analysis software toolboxes.

OpenNEURO[124] is an open-science neuroinformatics database that is freely available online. All researchers can browse and explore the public datasets, which have been shared by a wide range of global contributors. In addition, the OpenNEURO collaboration is committed to obtaining more public datasets compatible with brain imaging data structure (BIDS), a standardized format of neural imaging data. OpenNEURO is run by a research group led by Russell Poldrack and originated from the OpenfMRI project[124] in 2013. Today, the OpenNEURO database comprises MRI (17 940 participants, 509 public datasets), positron emission tomography (PET) (9 participants, 7 public datasets), magnetoencephalography (MEG) (365 participants, 21 public datasets), EEG (2 454 participants, 68 public datasets), and intracranial electroencephalography (iEEG) (202 participants, 12 public datasets).

The Allen Brain Map is a large, open science platform established by the Allen Institute for Brain Science, whose goal is to accelerate neuroscience research with the release of large-scale, publicly available atlases of the brain[38]. The Allen Institute publicly shares all the data, products, and findings from their research work. The main projects in the Allen Brain Map consist of the following databases. The Allen Brain Atlases capture patterns of gene expression across the brain in various species. The Allen Cell Types database contains the different types of neurons and other brain cells in human and mouse brains. Connectivity includes neural connections at scales ranging from the whole brain to the subcellular level. Finally, the Allen Brain Observatory contains the result of a series of systematic experiments conducted on mice with a variety of visual stimuli. Multiple recording modalities are the key features of this project, including calcium imaging response from multiple cortical visual areas across hundreds of two-photon imaging sessions and spiking activity from the visual cortex, hippocampus, and thalamus across dozens of electrophysiology sessions. In addition, a series of software toolkit resources, e.g., the AllenAPI and AllenSDK and modelling tools, are provided for related analysis work.

Collaborative Research in Computational Neuroscience (CRCNS) is a website primarily developed by Jeffrey L. Teeters of both of the Redwood Center for Theoretical Neuroscience at UC Berkeley[125] that hosts large-scale and high-quality experimental datasets. The data range from physiological recordings from sensory and memory systems to eye movement data. Specifically, datasets are classified according to their corresponding collection area for navigation, such as the visual cortex, auditory cortex, motor cortex, hippocampus, retina, and LGN.

Some software toolboxes have been developed as auxiliary resources for neuroscience researchers. NeuroRA is an easy-to-use toolbox based on Python, which can conduct representational similarity analysis (RSA) on nearly all kinds of neural data, including behavioral, EEG, MEG, stereoelectroencephalography (sEEG), electrocorticography (ECoG), fMRI and others. In addition, users can perform neural pattern similarity (NPS), classification-based EEG decoding in NeuroRA[126]. Brainlife provides an online, community-based platform where users can publish code and data while integrating cloud-computing resources to run their projects[127]. MultiVariate pattern analysis in Python (PyMVPA)[128] is a free Python package that provides a handy interface to a wide range of algorithms for classification, regression, feature extraction, data input and output, and other data processing demands in the neuroimaging field. Many popular machine learning packages, such as scikit-learn, shogun, the modular toolkit for data processing (MDP), etc., are integrated well into the PyMVPA framework[129, 130]. Recently, Huang et al.[131] developed an open-source toolbox for human brain mapping and decoding called Easy fMRI. This toolbox includes advanced machine learning techniques and high-performance computing for analyzing task-based fMRI datasets. It provides a friendly GUI-based environment for conducting feature analysis, hyperalignment, multi-voxel pattern analysis (MVPA), RSA, and more.

## 6 Open challenges and future directions

As we mentioned in Section 4 above, the issue of visual neural decoding has been a topic of interest for decades, with rapid advances in the development of both brain-activity recording techniques and neural decoding analysis methods. Here, we highlight several potential directions and open challenges and hope to provide researchers with insight into this issue.

Open challenges:

1) Most of the current visual decoding studies are offline, that is, the whole decoding process is limited by the scale of the recorded neural physiological data. These methods cannot ensure equivalent performance for new data as that for the test set (derived from the recorded neural data). In practice, medical engineering applications, such as brain-machine interfaces, aim to decode information that is usually detected by specific neuroimaging devices in real-time. Therefore, more challenges remain in online deployment for current state-of-the-art visual neural decoders. Researchers should consider online applicability when designing visual decoders.

2) Despite the availability of some public neuroscience resources, as introduced in Section 5 above, large public physiological datasets will remain an ongoing demand for a long time to come. The main reasons are the scarcity of public large-scale neural recording data and the high costs of physiological experiments in accessing these data.

3) To date, most visual neural decoders have been designed according to the specific neural recording modality available to researchers. This type of modality-specific neural decoder limits the generalization to different neural-decoder applications.

Future directions:

1) Given their strong capability for data fitting, machine learning techniques, especially the deep learning techniques, have greatly improved the performance of visual neural decoding systems, outperforming traditional methods, i.e., linear methods and unsophisticated Bayesian methods. The trajectory of neuroscience research will involve new deep learning techniques, given the rapid advancements in deep learning-based research fields such as computer vision, and the increasing speed of deep-learning and computationally friendly hardware.

2) The "transfer learning" strategy in the computer vision field, i.e., transfer of a model trained on one dataset to another problem (usually involving a smaller scale of data), provides a promising means to ameliorate the decoding performance in the field of computer vision. Some decoding studies have made use of this type of technique. For example, Shen et al.[117] used a comparator network trained on the ImageNet dataset[94] as part of their deep image reconstruction model from fMRI data. VanRullen and Reddy[123]. trained a VAE using a GAN over the public, large-scale face dataset CelebA and then mapped the recorded fMRI signal into the latent space in the VAE to achieve face reconstruction.

3) In addition, given the difficulties in large-scale pairwise neural signal communication, semisupervised learning techniques have been used to explore visual decoding without the need for a ground truth[132−136]. For example, Beliy et al.[132] leveraged a self-supervision technique to train their reconstruction model with unlabeled fMRI signals. Du et al.[134] solved the labelled-data-scarcity problem by casting the semisupervised classification problem as a specialized missing data imputation task.

4) Multimodality and cross-modal decoding are also future directions beyond reconstruction that have become popular in visual neural decoding studies. Multimodality decoding is the decoding of a combination of signals from more than one type of recording modality. For example, Ibayashi et al.[137] conducted speech decoding from signals in the ventral sensorimotor cortex, simultaneously leveraging information from spikes and local field potentials. Cross-modal decoding describes joint decoding models compatible with different recording modalities. For instance, Xu et al.[44] proposed a transcoding framework in which the stimuli can be reconstructed from spikes transcoded from signals from multi modalities.

5) Although several emerging open large-scale neural datasets have been made available (see Section 5), a great demand persists for standardized benchmarks of datasets and metrics in visual neural decoding, which could pave the way for strong baseline decoding models and widely recognized validation and evaluation standards for incoming studies in this field. Focusing on different fields, the Brain-Score platform[138], where submitted models can be scored on a range of brain benchmarks and new benchmarks can be incorporated to challenge the models, serves as a good example for reference in the development of visual learning decoding benchmarks.

6) Most of the current visual decoding studies are based on neural data collected from single brain areas, e.g., the V1 cortex, the LGN, and the retina. The interaction and association from the whole-brain connectivity are usually disregarded. Here, we suggest that researchers in the visual decoding field focus on the simultaneous study of neuroscience and brain science and regard visual neural decoding from a macro perspective. Recently, Kriegeskorte and Diedrichsen[139] proposed that the brain is not a conventional computer, but nothing a dynamic system in his review on brain coding. Haxby et al.[140] criticized decodability as a poor guide for revealing the content of neural representations in his decoding opinion. These debates and discussions deserve focus and in-depth consideration by neural decoding researchers.

## 7 Conclusions

In this paper, we first briefly analyzed the evolution of decoding tasks, i.e., classification, identification, and reconstruction, as this research field has developed. And we introduced the main neural recording modalities used in visual neural decoding and analyzed the characteristics of the data they acquire. Then we reviewed the main types of decoding approaches that researchers have proposed in recent decades in this field. Open data resources of data and toolkits, as well as open challenges and potential future directions of visual neural decoding, are suggested as well. The ultimate purpose of visual decoding is to decode the content of our experience in the absence of visual input. However, the scarcity of pairwise neurophysiological stimulus datasets and accurate, large-scale recording neural modalities continue to hinder the development of this discipline. Nevertheless, the importance of visual neural decoding cannot be understated. The development of neural decoding technology will promote the development of neural prostheses and brain-computer interface devices. We hope that our brief review will inspire ideas for future work in the cross-disciplinary field of brain science and neural computing.

## Acknowledgements

This work was supported by National Natural Science Foundation of China (Nos. 62176003 and 62088102), and the Royal Society Newton Advanced Fellowship of the UK (No. NAF-R1-191082).

## Open Access

This article is licensed under a Creative Commons Attribution 4.0 International License, which permits use, sharing, adaptation, distribution and reproduction in any medium or format, as long as you give appropriate credit to the original author(s) and the source, provide a link to the Creative Commons licence, and indicate if changes were made.

The images or other third party material in this article are included in the article's Creative Commons licence, unless indicated otherwise in a credit line to the material. If material is not included in the article's Creative Commons licence and your intended use is not permitted by statutory regulation or exceeds the permitted use, you will need to obtain permission directly from the copyright holder.

To view a copy of this licence, visit http://creativecommons.org/licenses/by/4.0/.
